# Simultaneous blockade of α1b-adrenergic and 5HT2A-serotonergic receptors for the treatment of alcohol use disorder: a randomized, placebo-controlled proof-of-concept phase-2 trial

**DOI:** 10.1192/j.eurpsy.2025.310

**Published:** 2025-08-26

**Authors:** J. Guiraud

**Affiliations:** 1 Psychiatry, Amsterdam UMC, University of Amsterdam, Amsterdam, Netherlands; 2 Vergio, Clichy, France

## Abstract

**Introduction:**

Alcohol use disorder (AUD) is associated with increased dopaminergic activity, and evidence suggests that α1b-adrenergic and 5HT2A serotonergic receptors play critical roles in modulating dopamine-mediated behavioral responses. Preclinical studies indicated that simultaneous blockade of these receptors with prazosin (α1b blocker) and cyproheptadine (5HT2A blocker) can reduce alcohol preference. This phase 2 clinical study aimed to evaluate the efficacy and safety of this combination in reducing alcohol consumption among patients with severe AUD.

**Objectives:**

The primary objective of the study was to assess the efficacy and safety of a combination of prazosin extended-release (ER) and cyproheptadine in reducing total alcohol consumption (TAC) in patients with severe AUD.

**Methods:**

This was a phase 2, double-blind, parallel-group, placebo-controlled, randomized clinical trial conducted across 32 addiction treatment centers in France. A total of 154 participants (108 men and 46 women) with severe AUD were randomly assigned to one of three treatment groups for 3 months: 1) low-dose group (LDG) with 8 mg cyproheptadine and 5 mg prazosin ER daily, 2) high-dose group (HDG) with 12 mg cyproheptadine and 10 mg prazosin ER daily, or 3) placebo group (PG). The primary outcome was the change in TAC from baseline to Month 3. Secondary outcomes included changes in heavy drinking days, abstinence days, Obsessive Compulsive Drinking Scale (OCDS), and Beck Depression Inventory (BDI) scores. Safety was assessed through adverse events (AEs), sedation, and orthostatic hypotension (OH).

**Results:**

A significant main treatment effect in TAC change was observed in the intent-to-treat (ITT) population (p=0.039). Compared to the placebo group, the HDG showed a greater reduction in TAC from baseline to Month 3 (-23.6 g/day, p=0.016, Cohen’s d=-0.44), while the LDG also showed a reduction (-18.4 g/day, p=0.048). In the very high-risk drinking level subgroup (>100g/day of pure alcohol for men and >60g/day for women), the HDG showed a reduction of -29.8 g/day compared to the PG (p=0.031, d=-0.51). A significant dose-response relationship (p=0.027) was observed. Both low and high doses were well-tolerated, with AEs predominantly mild or moderate. No serious adverse events were reported in the HDG, and OH incidence was comparable across groups.

**Conclusions:**

The combination of prazosin and cyproheptadine showed efficacy in reducing alcohol consumption in individuals with severe AUD, with a larger effect observed in the high-dose group. Both doses were well-tolerated, with a safety profile comparable to placebo. These findings suggest that the prazosin-cyproheptadine combination may be a promising treatment option for severe AUD and warrant further investigation in phase 3 trials.

**Disclosure of Interest:**

J. Guiraud Shareolder of: Vergio, Employee of: Vergio

